# Pullulan Based Bioconjugates for Ocular Dexamethasone Delivery

**DOI:** 10.3390/pharmaceutics13060791

**Published:** 2021-05-26

**Authors:** Eva Kicková, Stefano Salmaso, Francesca Mastrotto, Paolo Caliceti, Arto Urtti

**Affiliations:** 1 Department of Pharmaceutical and Pharmacological Sciences, University of Padova, via F. Marzolo 5, 35131 Padova, Italy; eva.kickova@studenti.unipd.it (E.K.); stefano.salmaso@unipd.it (S.S.); francesca.mastrotto@unipd.it (F.M.); 2 Faculty of Health Sciences, School of Pharmacy, University of Eastern Finland, Yliopistonranta 1C, 70211 Kuopio, Finland; 3 Drug Research Program, Faculty of Pharmacy, University of Helsinki, Viikinkaari 5 E, 00710 Helsinki, Finland

**Keywords:** pullulan, dexamethasone, hydrazone, ocular drug delivery, controlled release

## Abstract

Posterior segment eye diseases are mostly related to retinal pathologies that require pharmacological treatments by invasive intravitreal injections. Reduction of frequent intravitreal administrations may be accomplished with delivery systems that provide sustained drug release. Pullulan-dexamethasone conjugates were developed to achieve prolonged intravitreal drug release. Accordingly, dexamethasone was conjugated to ~67 kDa pullulan through hydrazone bond, which was previously found to be slowly cleavable in the vitreous. Dynamic light scattering and transmission electron microscopy showed that the pullulan-dexamethasone containing 1:20 drug/glucose unit molar ratio (10% *w*/*w* dexamethasone) self-assembled into nanoparticles of 461 ± 30 nm and 402 ± 66 nm, respectively. The particles were fairly stable over 6 weeks in physiological buffer at 4, 25 and 37 °C, while in homogenized vitreous at 37 °C, the colloidal assemblies underwent size increase over time. The drug was released slowly in the vitreous and rapidly at pH 5.0 mimicking lysosomal conditions: 50% of the drug was released in about 2 weeks in the vitreous, and in 2 days at pH 5.0. In vitro studies with retinal pigment epithelial cell line (ARPE-19) showed no toxicity of the conjugates in the cells. Flow cytometry and confocal microscopy showed cellular association of the nanoparticles and intracellular endosomal localization. Overall, pullulan conjugates showed interesting features that may enable their successful use in intravitreal drug delivery.

## 1. Introduction

Ocular drug delivery is a major challenge in drug development [1,2]. In particular, efficient delivery to the retina and other posterior tissues of the eye is difficult to be achieved, requiring invasive intravitreal injections. Drug delivery systems providing for prolonged drug release may overcome frequent injections and reduce pain and complications such as risk of infections or tissue damage.

Even though synthetic and natural polymers have been widely used to produce ophthalmic drug delivery systems either for topical or intravitreal applications, polymer conjugates have not been extensively investigated [3,4]. Nevertheless, polymer conjugates may be easily injected into the vitreous, providing for prolonged residence time, slow drug release, and possible cell targeting.

The polymer shape, size, charge and water solubility are important features for intravitreal administration of polymer-based drug carriers. Vitreous humor contains a 3D gel-like matrix of collagen and hyaluronic acid with mesh size of 500–550 nm [2,5,6,7]. Thus, diffusion of macromolecules and colloidal systems is slowed down in the vitreous [8]. Neutral or negatively charged intravitreal carriers can diffuse in the vitreous, thus reaching the retina and other structures affected by specific misfunctions. In contrast, cationic systems are preferentially trapped in the vitreous network by electrostatic interactions with its components [4,9], where they can slowly release the drug. In this realm, supramolecular polysaccharide-drug conjugates are interesting systems for intravitreal drug delivery as they can be chemically manipulated by conjugation strategies to tailor their biopharmaceutical properties. Accordingly, polysaccharide conjugates can be designed to yield prolonged residence time, extended drug release and cell targeting.

Pullulan is an interesting platform to produce conjugates for drug delivery. This polysaccharide produced by *Aureobasidium pullulans* [10,11] possesses in fact the main requisites for invasive administration: biodegradability, water solubility and biocompatibility [10,11,12,13]. Furthermore, pullulan can be properly derivatized to conjugate drugs or other chemical moieties along the polymer backbone to yield colloidal systems for controlled drug release. For example, pullulan derivatization with cholesterol was found to self-assemble into nanoparticles that can physically entrap drugs. Paclitaxel and doxorubicin were conjugated to pullulan to yield self-assembling derivatives that release the drugs according to controlled behavior [14,15,16]. Pullulan derivatized with PresS1 peptide and doxorubicin was found to target hepatocarcinoma cells, resulting in selective chemotherapy [17].

The chemical procedure for drug conjugation to the polymer backbone is paramount to control the drug release rate. Recently, we showed that the release rate of conjugated drug from pullulan can be controlled by selecting proper cleavable linkers [18]. In particular, hydrazone linker was found to provide slow drug release under neutral conditions resembling the vitreous compartment, whereas previous studies show that hydrazone is rapidly cleaved under acidic conditions. For this reason, a variety of bioconjugates have been obtained by conjugating anticancer drugs to natural or synthetic polymer through hydrazone bond [19,20,21,22,23,24,25]. Nevertheless, these bioconjugates have been shown to be fairly stable in plasma, by virtue of the higher linker stability under physiological pH, suggesting that they can be properly exploited to yield slow drug release in the vitreous.

We report here the development of a new pullulan-dexamethasone conjugate as a potential intravitreal drug delivery system. Dexamethasone was selected as a model drug since it is a hydrophobic corticosteroid used as anti-inflammatory and immune suppressing drug in ophthalmology [26,27,28,29]. A synthetic procedure for pullulan activation and dexamethasone conjugation is reported. Pullulan-dexamethasone showed prolonged drug release in the vitreous as a result of the slow cleavage of the hydrazone linker used to conjugate the drug to the polysaccharide backbone.

## 2. Materials and Methods

Pullulan (67 kDa) was supplied by Hayashibara Biochemical Laboratories (Okayama, Japan). Dexamethasone, hydrazine hydrate, sodium hydride, ethyl bromoacetate, trifluoroacetic acid, triethylamine, 4-chloro-3-nitrobenzoic acid, picrylsulfonic acid solution (TNBS, 1 M in H_2_O), Tween^®^ 80, *N*,*N*-dimethylformamide (DMF, anhydrous), Dimethyl sulfoxide (DMSO, anhydrous), deuterium oxide (D_2_O) and dimethyl sulfoxide-*d_6_* (DMSO-*d_6_*) were obtained from Sigma/Merck KGaA (Darmstadt, Germany). Cyanine3-NHS ester was purchased from Lumiprobe GmbH (Hannover, Germany). Dulbecco’s Modified Eagle’s Medium/nutrient mixture F-12 (DMEM/F12 medium) without glutamine was purchased from Aurogene (Rome, Italy). Penicillin-Streptomycin solution (10,000 units penicillin and 10 mg streptomycin/mL), L-glutamin (200 mM), Trypsin (10×), 3-(4,5-Dimethylthiazol-2-yl)-2,5-diphenyltetrazolium bromide (MTT), Fetal Bovine Serum (FBS), and sterile phosphate buffered saline (PBS, 1X) were obtained from Sigma (St Louis, MO, USA). 1 CD107a (LAMP-1) monoclonal antibody was purchased from Thermo Fisher Scientific Inc./Invitrogen (Carlsbad, CA, USA). EEA1 antibody was purchased from Cell Signaling Technology, Inc. (Danvers, MA, USA). Goat anti-rat and anti-rabbit IgG H&L (Alexa Fluor^®^ 488) were purchased from Abcam plc. (Cambridge, UK). 4′,6-diamidino-2-phenylindole (DAPI) was purchased from Vector laboratories, Inc. (Burlingame, CA, USA). The water used for all experiments was produced with the Millipore Milli-Q purification system (Burlington, MA, USA), filtered and sterilized. Anhydrous DMF was kept under molecular sieves 24 h before using. All chemicals used in this study were at high analytical grade. Vitreous humor was obtained from porcine eyes, homogenized and provided by University of Eastern Finland (Kuopio, Finland).

### 2.1. Synthesis of Pullulan-Dexamethasone

Pullulan-dexamethasone was obtained according to a three-step procedure: 1. synthesis of carboxyethyl-pullulan (Section 2.1.1), 2. conversion of carboxyethyl-pullulan into carboxyhydrazide-pullulan (Section 2.1.2) and 3. conjugation of dexamethasone (Section 2.1.3).

#### 2.1.1. Synthesis of Carboxyethyl-Pullulan

Pullulan was functionalized using a modified protocol reported in the literature [24]. Pullulan (3 g, 18.5 mmol glucopyranose units, GPU) was dissolved in 150 mL anhydrous dimethylsulfoxide (DMSO) at 40 °C and slowly added of NaH (0.3 g, 12.5 mmol, 60% dispersion in mineral oil) under stirring. After 1 h stirring at 40 °C, 6.6 mL ethyl bromoacetate (9.9 g, 59.3 mmol) in 21 mL anhydrous DMSO was added dropwise to the reaction mixture under stirring and the reaction was maintained at 40 °C for 7 h and then at room temperature for 2 days. Cold water was slowly added to quench the reaction. The polymer was isolated by precipitation in 400 mL acetone and 5 min centrifugation at 4000 rpm. The material was dried under vacuum, redissolved in 15 mL water and dialyzed by using MWCO 3.5–5 kDa Spectra/Por^®^ membrane (Carl Roth GmbH + Co. KG, Karlsruhe, Germany) against 5 L of deionized (DI) water for 16 h. The dialyzed solution was freeze-dried and carboxyethyl-pullulan was obtained as a white powder with 80% mol/mol recovery yield. The product was analyzed by FT-IR (FT-IR-6000 Jasco, Tokyo, Japan), elemental analysis (varioMICRO V4.0.10 instrument at CHNS Mode; Elementar Analysensysteme GmbH, Hanau, Germany), ^1^H NMR and ^13^C NMR (Bruker DPX400 and DMX600, Billerica, MA, USA), which showed 15% GPU carboxyethyl group derivatization yield (Supplementary Information, SI-2).

FT-IR (KBr). 3402 (-OH), 2930 (C-H), 1736 (O-C=O), 1638 (C-O-C) cm^−1^.
Elemental analysis. Calcd: C, 45.02, H, 6.32%. Found: C, 40.96%; H, 6.15%.^1^H NMR (400 MHz, D_2_O). δ 5.45 (d, 1H, (1→4)-α-glycosidic bond), 5.00 (s, 1H, (1→6)-α-glycosidic bond), 4.56–3.27 [5H, remaining Hs of glucopyranose; 4H, 2x -CH_2_-, carboxyethyl group], 1.33 (t, 3H, -CH_3_, carboxyethyl group).
^13^C NMR (151 MHz, D_2_O). δ 172.32 (s, C=O, carboxyethyl group), 100.21, 99.74, 97.90, 80.11 (s, -CH_2_-, carboxyethyl group), 77.76, 77.14, 73.54, 73.41, 73.31, 73.04, 72.69, 71.69, 71.58, 71.48, 71.38, 71.31, 71.14, 70.68, 70.31, 70.06, 69.70, 69.44, 69.32, 68.43 (s, -CH_2_-, carboxyethyl group), 66.44, 62.33, 60.67, 60.37, 13.31 (s, -CH_3_, carboxyethyl group).

#### 2.1.2. Synthesis of Carboxyhydrazide-Pullulan

Carboxyl groups of carboxyethyl-pullulan were amidated with hydrazine to yield hydrazides according to the adapted protocol reported in the literature [24]. Carboxyethyl-pullulan (2.5 g, 14.3 mmol GPU) was dissolved in 100 mL anhydrous DMSO and added dropwise in 1 h of 2.5 mL hydrazine hydrate (2.6 g, 80.3 mmol). The reaction mixture was stirred at room temperature for 2 days. The product was isolated using the same procedure reported above (Section 2.1.1). The freeze-dried carboxyhydrazide-pullulan was obtained as a white powder with 94% mol/mol recovery yield. The product was analyzed by FT-IR, elemental analysis, ^1^H NMR and ^13^C NMR, which showed 15% GPU derivatization yield (-NHNH_2_). The hydrazide group analysis performed by TNBS assay [30] using a calibration curve obtained with hydrazine yielded 14.8% GPU derivatization.

FT-IR (KBr). 3400 (-OH), 2929 (C-H), 1655 (N-C=O), 1638 (C-O-C) cm^−1^.
Elemental analysis. Calcd: C, 43.55%, H, 6.15%, N, 2.42%. Found: C, 40.78%; H, 6.53%; N, 1.89%.
^1^H NMR (400 MHz, D_2_O). δ 5.42 (d, ^1^H, (1→4)-α-glycosidic bond), 5.00 (s, 1H, (1→6)-α-glycosidic bond), 4.53–3.36 [5H, remaining Hs of glucopyranose; 2H, -CH_2_-, carboxyethyl group].
^13^C NMR (101 MHz, D_2_O). δ 170.84 (s, C=O, carboxyethyl group), 100.25, 99.73, 97.96, 80.24 (s, -CH_2_-, carboxyethyl group), 77.87, 77.35, 73.81, 73.48, 73.32, 73.08, 72.52, 71.74, 71.63, 71.52, 71.43, 71.34, 71.20, 71.03, 70.36, 70.16, 69.79, 69.51, 69.38, 66.33, 62.37, 60.71, 60.44.

#### 2.1.3. Synthesis of Pullulan-Dexamethasone

Pullulan-dexamethasone was obtained by conjugating the carbonyl group of dexamethasone to carboxyhydrazide-pullulan through a hydrazone bond under acidic catalyzed conditions using an adapted method reported in the literature [31]. Carboxyhydrazide-pullulan (0.29 g, 1.7 mmol GPU) was dissolved in 55 mL of 6:1 *v*/*v* dimethylformamide (DMF):DMSO anhydrous mixture and added of 97 μL trifluoroacetic acid (TFA, 1.26 mmol). The reaction mixture was stirred at 40 °C for 3 h. Dexamethasone (0.12 g, 0.3 mmol) in 1 mL of 6:1 *v*/*v* DMF:DMSO anhydrous mixture was added to the reaction mixture maintained under nitrogen atmosphere and stirred at 40 °C for 2 days in the dark. At scheduled times, 150 μL aliquots were withdrawn and analyzed by RP-HPLC to assess consumption of free dexamethasone during the conjugation progress and by TNBS assay to assess the conjugation of the hydrazide groups (SI-3, Figure S1). The polymer was then isolated by precipitation in 150 mL dichloromethane (DCM) and 5 min centrifugation at 14,000 rpm. The solid residue was washed two times with 100 mL DCM, recovered by centrifugation, dissolved in DMF and again isolated by precipitation in 100 mL DCM followed by centrifugation and drying under vacuum to remove traces of organic solvents. The white solid pullulan-dexamethasone was obtained with 90% mol/mol recovery yield and analyzed by FT-IR and ^1^H NMR. The use of 4-chloro-3-nitrobenzoic acid as internal standard in the ^1^H NMR analysis provided for determination of the degree of dexamethasone conjugation, which resulted in be 5.2% derivatized GPU yield (corresponding to 10% *w*/*w* conjugation, SI-4).

FT-IR (KBr). 3408 (-OH), 2926 (C-H), 1654 (-C=O-NH-N=C), 1637 (C-O-C) cm^−1^.
^1^H NMR (400 MHz, D_2_O). δ 7.57 (d, 1H*ar*, aromatic proton, dexamethasone), 6.47 (d, 1H*ar*, dexamethasone), 6.27 (s, 1H*ar*, dexamethasone), 5.41 (d, 1H, (1→4)-α-glycosidic bond), 4.99 (s, 1H, (1→6)-α-glycosidic bond), 4.60–3.40 (5H, remaining Hs of glucopyranose; 2H, -CH_2_-, carboxyethyl group; 12H, remaining Hs of dexamethasone), 1.59 (s, 3H, -CH_3_*ar*, dexamethasone), 1.02 (s, 3H, -C-CH_3_, dexamethasone), 0.92 (d, 3H, -CH-CH_3_, dexamethasone).
^1^H NMR (400 MHz, DMSO-*d_6_*, with internal standard 4-chloro-3-nitrobenzoic acid). δ 8.47 (d, 1H*ar*, IS), 8.16 (dd, 1H*ar*, IS), 7.95 (d, 1H*ar*, dexamethasone), 7.88 (d, 1H*ar*, IS), 5.65–2.99 [1H, (1→4)-α-glycosidic bond; 1H, (1→6)-α-glycosidic bond; 5H, remaining Hs of glucopyranose; 2H, -CH_2_-, carboxyethyl group; 12H, remaining Hs of dexamethasone], 1.24 (s, 3H, -CH_3_*ar*, dexamethasone), 1.17–1.00 (m, 3H, -C-CH_3_, dexamethasone), 0.97–0.74 (m, 3H, -CH-CH_3_, dexamethasone).

### 2.2. Synthesis of Pullulan-Dexamethasone-Cyanine3

Pullulan-dexamethasone labeling with cyanine3 was performed according to published protocols [27]. Briefly, pullulan-dexamethasone (0.3 g, 1.7 mmol GPU) was dissolved in 65 mL of 1:2 *v*/*v* DMF:DMSO anhydrous mixture followed by addition of 0.02 mL triethylamine (TEA, 0.14 mmol) and maintained at room temperature for 2 h. Cyanine3-NHS ester (0.007 g, 0.01 mmol) dissolved in 1 mL of anhydrous DMSO was added to the pullulan-dexamethasone solution. The reaction was maintained under nitrogen atmosphere and stirring at room temperature for 2 days in the dark. The conjugate was isolated by precipitation and washed with DCM as reported above (Section 2.1.3) to eliminate unconjugated cyanine3. The precipitate was dispersed in water, dialyzed against mQ water and freeze-dried. Pullulan-dexamethasone-cyanine3 was obtained as a pink powder with 92% mol/mol recovery yield and analyzed by ^1^H NMR, which showed 5.2% GPU dexamethasone conjugation yield and 1.1% GPU cyanine3 conjugation yield.

^1^H NMR (400 MHz, DMSO-*d_6_*, with internal standard (4-chloro-3-nitrobenzoic acid). δ 8.33 (d, 1H*ar*, IS), 8.07 (dd, 1H*ar*, IS), 7.67 (d, 1H*ar*, IS), 5.67–2.99 [1H, (1→4)-α-glycosidic bond; 1H, (1→6)-α-glycosidic bond; 5H, remaining Hs of glucopyranose; 2H, -CH_2_-, carboxyethyl group; 12H, remaining Hs of dexamethasone; 10H, 5x -CH_2_-, cyanine3], 2.67 (t, 3H, -CH_3_, cyanine3), 2.32 (q, 3H, -CH_3_, cyanine3), 1.24 (s, 3H, -CH_3_*ar*, dexamethasone), 1.18–1.03 (m, 3H, -C-CH_3_, dexamethasone), 0.96–0.73 (m, 3H, -CH-CH_3_, dexamethasone).

### 2.3. Synthesis of Pullulan-Cyanine3

Carboxyhydrazide-pullulan (0.17 g, 0.98 mmol GPU) was dissolved in 7 mL of anhydrous DMSO. 0.03 mL of cyanine3-NHS ester (0.003 g, 0.005 mmol) solution in anhydrous DMSO was added to the mixture under stirring in the dark followed by the addition of 0.01 mL TEA (0.08 mmol). The reaction mixture was stirred at room temperature for 2 days in the dark. The polymer was isolated by precipitation in 100 mL DCM and 5 min centrifugation at 14,000 rpm. The material was washed three times with 60 mL DCM and recovered by centrifugation. The crude precipitate was dispersed in 18 mL of mQ water, dialyzed against water and freeze-dried. The pink solid pullulan-cyanine3 was obtained with 88% mol/mol recovery yield. The ^1^H NMR analysis showed 3.1% GPU cyanine3 conjugation yield.

^1^H NMR (400 MHz, D_2_O). δ 7.36 (s, 1H*ar*, cyanine3), 5.43 (d, 1H, (1→4)-α-glycosidic bond), 5.00 (s, 1H, (1→6)-α-glycosidic bond), 4.60–3.40 [5H, remaining Hs of glucopyranose; 2H, -CH_2_-, carboxyethyl group; 10H, 5x -CH_2_-, cyanine3], 2.31 (s, 3H, -CH_3_, cyanine3), 2.09 (s, 3H, -CH_3_, cyanine3), 1.73 (s, 6H, 2x -CH_3_, cyanine3).
^1^H NMR (400 MHz, DMSO-*d_6_*, with internal standard (4-chloro-3-nitrobenzoic acid). δ 8.48 (d, 1H*ar*, IS), 8.17 (dd, 1H*ar*, IS), 7.90 (d, 1H*ar*, IS), 6.63 (d, 1H*ar*, cyanine3), 5.70–2.84 [1H, (1→4)-α-glycosidic bond; 1H, (1→6)-α-glycosidic bond; 5H, remaining Hs of glucopyranose; 2H, -CH_2_-, carboxyethyl group; 10H, 5x -CH_2_-, cyanine3], 2.67 (t, 3H, -CH_3_, cyanine3), 2.33 (q, 3H, -CH_3_, cyanine3).

Pullulan-dexamethasone, pullulan-dexamethasone-cyanine3 and pullulan-cyanine3 were dissolved in mQ water at 10 mg/mL concentration and the samples were treated in an orbital mixer at 100 rpm for 16 h in the dark. The stock samples were diluted when required.

### 2.4. Gel Permeation Chromatography

Gel permeation chromatography (GPC) analysis was carried out using two TSK gel columns in series (G4000 PWXL 10 μm, 7.8 × 300 mm and G3000 PWXL 7 μm, 7.8 × 300 mm, TOSOH Bioscience GmbH, Stuttgart, Germany) operated with a VISCOTEK TDA 302 instrument with triple detector array, refractive index, right angle light scattering, low angle light scattering, viscometer—DP (Malvern Instruments Ltd., Worcestershire, UK). Chromatographic analyses of 100 μL pullulan and pullulan derivative samples (4 mg/mL in 0.4 M ammonium acetate, pH 4.5) were performed at 40 °C by using 0.4 M ammonium acetate, pH 4.5, eluent and flow rate of 0.6 mL/min. The calibration curve was obtained by using GPC standards of pullulan and dextran.

### 2.5. Size and Zeta Potential Analyses

Aqueous solutions of pullulan and pullulan derivatives (1 mg/mL) were analyzed by dynamic light scattering (DLS) using the Zetasizer Nano ZS (Malvern Instrument Ltd., Malvern, UK). Zeta potential analyses were performed with 1 mg/mL polymer solutions in 1 mM phosphate buffer, pH 7.4. Pullulan-dexamethasone solutions (1 mg/mL) in 10 mM phosphate buffer, 150 mM NaCl (PBS), pH 7.4 incubated at three different temperatures (4, 25 and 37 °C), and in the homogenized vitreous (1:1 *v*/*v* homogenized vitreous: PBS) at 37 °C for 42 days in the dark were analyzed with Zetasizer Nano ZS at scheduled times. All analyses were performed in triplicate.

### 2.6. Transmission Electron Microscopy (TEM)

Five microliters of 1 mg/mL pullulan derivatives in mQ water were placed on a 400-mesh holey film grid. After 2 min, the excess of the water was removed by filter paper and the sample was stained with 1% *w*/*v* uranyl acetate in mQ water. The excess of staining solution was carefully removed by filter paper. The samples were imaged with the Tecnai G2 microscope instrument (FEI™, Hillsboro, OR, USA) operating at 100 kV. Images were captured with a Veleta (Olympus Soft Imaging System) digital camera.

### 2.7. Dexamethasone Release

One milliliter of a 5 mg/mL pullulan-dexamethasone solution in 1) PBS, pH 7.4; 2) 10 mM phosphate, 24 mM citric acid, 150 mM NaCl, pH 5.0; and 3) 1:1 *v*/*v* homogenized vitreous: PBS, pH 7.4, were dialyzed against 6 mL receiving medium by using Float-A-Lyzer devices (Spectra-Por^®^ Float-A-Lyzer^®^ G2, black, 1 mL, MWCO 3.5–5 kDa, Sigma-Aldrich, Co., St Louis, MO, USA). The receiving medium was the same used for preparation of the pullulan-dexamethasone samples supplemented with 1% *v*/*v* Tween 80 to enhance the drug solubility (SI-5, Figure S2). The receiving medium for samples containing vitreous was PBS, pH 7.4 supplemented with 1% *v*/*v* Tween 80. The dialysis was performed at 37 °C and the receiving medium was collected at selected time-points and replaced with fresh medium. The collected samples were analyzed by RP-HPLC using a Phenomenex Luna C18 column (5 μm, 100 Å, 250 × 4.60 mm, Torrance, CA, USA) eluted at 1 mL/min flow rate with acetonitrile (solvent A) and 50 mM phosphate buffer, pH 6.8 (solvent B) both added in a gradient mode: 0–3 min solvent A 30%, 3–12 min solvent A 30–90%, 13–14 min solvent A 90–95%, 15–25 min solvent A 95–30%. The UV detector wavelength was set at 240 nm for detection of dexamethasone. The amount of dexamethasone in the receiving media was assessed by using a titration curve obtained with dexamethasone solutions at known concentrations.

The drug release method was preliminarily validated by testing the diffusion of free dexamethasone at a concentration of 13.5 μg/mL in PBS, pH 7.4 and in 1:1 *v*/*v* homogenized vitreous: PBS, pH 7.4 from the Float-A-Lyzer system using the same protocols applied for the pullulan-dexamethasone conjugate.

The chemical identity of dexamethasone eluted from RP-HPLC was confirmed by ESI-TOF mass spectrometry using the Waters Xevo G2S Q-Tof mass spectrometer (Waters Corp., Milford, MA, USA). The dexamethasone elution volumes at 10–11 min from the RP-HPLC analyses were analyzed by mass spectrometer in positive ionization mode. The optimized instrument conditions for analysis of dexamethasone were: capillary voltage of 2 kV, sampling cone voltage of 40 V, desolvation temperature of 150 °C and source temperature of 120 °C. The theoretical mass of dexamethasone was simulated by the software program and compared with the obtained data.

### 2.8. Cytotoxicity Studies

Cell viability was assessed in vitro using ARPE-19 cells grown in DMEM/F12 medium containing 2 mM L-glutamine, 100 IU/mL penicillin and 100 μg/mL streptomycin supplemented with 10% *v*/*v* FBS (complete medium). The cells were grown in a humidified 5% CO_2_ atmosphere at 37 °C. The cells were incubated with: 1. free dexamethasone in 0–100 μM concentration range; 2. with pullulan-dexamethasone or pullulan-dexamethasone-cyanine3 at 0–400 μM dexamethasone equivalent concentrations; and 3. with free pullulan at concentrations used for pullulan-dexamethasone derivatives (0–1.6 mg/mL of polymer). All samples were prepared in pure DMEM/F12 medium. Two incubation conditions were applied in this study: 1.2 × 10^4^ cells/well in a 96-well plate, grown for 24 h and incubated for 24 h with dexamethasone, or pullulan-dexamethasone derivatives, or pullulan, in FBS-free complete medium; and 2.8 × 10^3^ cells/well in a 96-well plate, grown for 24 h and incubated for 24 h with FBS-free complete medium containing: 0–400 μM dexamethasone; 0–1.41 mg/mL pullulan; 0–1.57 mg/mL pullulan-dexamethasone derivatives (corresponding to 0–400 μM of dexamethasone and 0–1.41 mg/mL pullulan); 2.2 mg/mL pullulan-dexamethasone-cyanine3 (corresponding to 0–400 μM of dexamethasone and 0–1.41 mg/mL pullulan). Test material exposure to the cells was followed by removal of medium, replacement with fresh complete medium and cell culture for further 48 h.

After incubation, the media were discharged, the cells were washed three times with 100 μL sterile Ca^2+^ and Mg^2+^ free PBS, pH 7.4, and then incubated with 200 μL/well MTT solution in DMEM/F12 for 3 h in a humidified 5% CO_2_ atmosphere at 37 °C. The solution was removed and added of 200 μL/well DMSO. The 96-well plate was incubated at room temperature in the dark under gentle shaking for 30 min. The absorbance was read by spectrophotometric analysis at 570 nm using a Varioskan™ LUX plate reader (Thermo Fisher Scientific, Madison, WI, USA). The test was performed in triplicate and analysis of cell viability was derived as percentage of untreated cells as a control.

### 2.9. Flow Cytometric Analysis

ARPE-19 cells were seeded at a density of 2 × 10^5^ cells/well in a 24-well plate in complete medium (250 μL) and grown for 24 h in a humidified 5% CO_2_ atmosphere at 37 °C. The medium was then discharged, the cells were washed with sterile Ca^2+^ and Mg^2+^ free PBS, pH 7.4 (3 × 200 μL) and incubated with pullulan-dexamethasone-cyanine3 at 0–225 μM equivalent concentration of conjugated dexamethasone dissolved in pure DMEM/F12 medium (250 μL). The cells were incubated for 1 h in a humidified 5% CO_2_ atmosphere at 37 °C. Then, the solutions were discharged, the cells were washed with sterile Ca^2+^ and Mg^2+^ free PBS, pH 7.4 supplemented with 10% *v*/*v* FBS (2 × 400 μL) to remove adsorbed polymer on the cell surface and plastics and with Ca^2+^ and Mg^2+^ free PBS, pH 7.4 (1 × 150 μL). Cells were detached from wells by 4 min treatment with 200 μL/well of 0.125 mg/mL trypsin solution in Ca^2+^ and Mg^2+^ free PBS, pH 7.4. The cells were transferred into FACS tubes containing 100 μL of FACS washing buffer (PBS, pH 7.4 containing 0.5% *w*/*v* bovine serum albumin, 5 mM EDTA and 2 mM NaN_3_) and 100 μL of PFA (PBS, pH 7.4 containing 4% *w*/*v* PFA). The tubes were stored at 4 °C in the dark and analyzed with a BD FACS CANTO™ II, operated by FACSDIVA software (BS Biosciences, San Jose, CA, USA) with laser set at λ_ex_ 570 nm for cyanine3 detection. Data were analyzed by Flowing Software version 2.5.1 (By Perttu Terho, Turku Centre for Biotechnology University of Turku, Finland, in collaboration with Turku BioImaging).

All graphical interpretations of the obtained data were prepared by using a 1992–2017 GraphPad Software, Inc. Prism 7 for Windows, Version 7.04.

### 2.10. Confocal Microscopy

ARPE-19 cells were seeded on 13 mm^2^ glasses cover dishes at a density of 0.8 × 10^5^ cells/well a 24-well plate in 250 μL complete medium and grown in a humidified 5% CO_2_ atmosphere at 37 °C. After 24 h, the medium was discharged, the cells were washed three times with 200 μL sterile Ca^2+^ and Mg^2+^ free PBS, pH 7.4, and incubated with 0.2 mg/mL of pullulan-dexamethasone-cyanine3 in 250 μL DMEM/F12 medium without FBS in a humidified 5% CO_2_ atmosphere at 37 °C. After 1 h, cells were washed two times with 400 μL sterile Ca^2+^ and Mg^2+^ free PBS, pH 7.4, containing 10% *v*/*v* FBS to remove adsorbed polymer on the cell surface and plastics and once with 150 μL sterile Ca^2+^ and Mg^2+^ free PBS, pH 7.4. The cells were fixed with 4% *w*/*v* PFA solution in PBS, pH 7.2 for 20 min. The cell membrane was pre-treated by PBS, pH 7.4 containing 0.5% *v*/*v* Tween 20 and 4% *w*/*v* bovine serum albumin. After 10 min, the cells were incubated with primary antibody rabbit anti-EEA1 (dilution 1:100) and secondary antibody Alexa Fluor 488 labeled goat anti-rabbit IgG (dilution 1:200) for endosome staining or with primary antibody rat anti-mouse anti-LAMP1 (dilution 1:100) and secondary antibody Alexa Fluor 488 labeled goat anti-rat IgG (dilution 1:200) for lysosome staining. Each antibody incubation was performed for 1 h. The cell nuclei were stained with 2 μg/mL of DAPI in PBS, pH 7.4, for 10 min. Finally, the dishes were mounted on microscope slides using Mowiol^®^ 4-88 (Sigma-Aldrich, Co., St Louis, MO, USA), prepared as 10% *w*/*v* solution in a 1:3.8 glycerol/0.13 M Tris-HCl buffer pH 8.5 *v*/*v* ratio mixture) as mounting medium. Images were collected using confocal laser scanning microscope ZEISS LSM 800 (Carl Zeiss Microscopy GmbH, Oberkochen, Germany). Lasers were set at 405 nm for DAPI, 488 nm for Alexa Fluor 488, and 561 nm for cyanine3 detection, and Zen Pro 2.3 software was used for image acquisition. The images were processed with ImageJ software 1.52n (National Institutes of Health software package).

## 3. Results and Discussion

The starting material, pullulan, underwent a preliminary characterization by gel permeation chromatography (GPC) to assess the molecular weight and polydispersity, which resulted in be 67 kDa and 3 M_w_/M_n_, respectively.

### 3.1. Synthesis of Bioconjugates

**Scheme 1:**
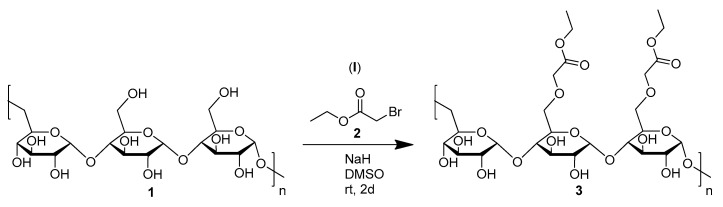

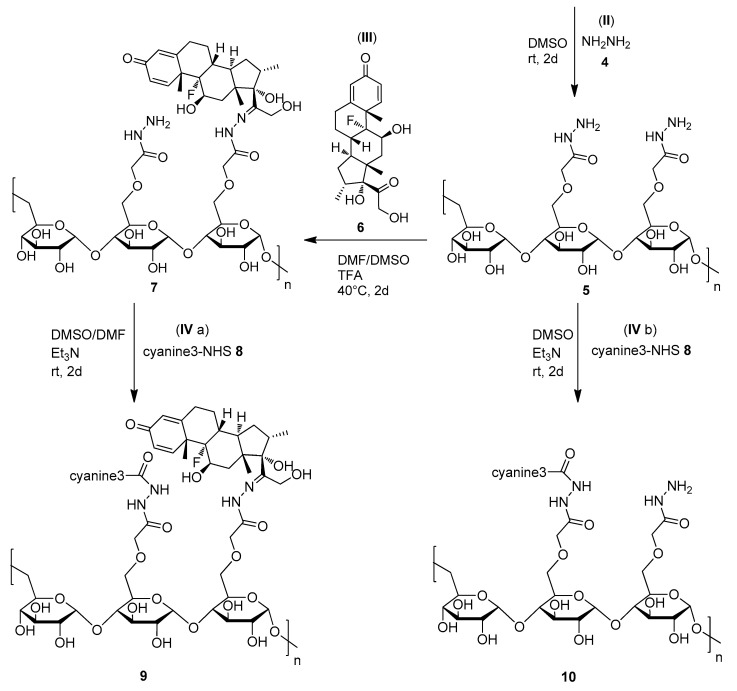
Schematic representation of pullulan-dexamethasone derivative synthesis. *Reagents and conditions*: (**I**) ethyl bromoacetate **2**, sodium hydride (NaH), anhydrous DMSO, room temperature (rt), two days (2d); (**II**) hydrazine hydrate **4**, anhydrous DMSO, room temperature (rt), two days (2d); (**III**) dexamethasone **6**, anhydrous DMF:DMSO, trifluoroacetic acid TFA, 40 °C, 2 days (2d); (**IV** a) NHS activated cyanine3 **8**, anhydrous DMF:DMSO, TEA, room temperature (rt), two days (2d); (**IV** b) NHS activated cyanine3 **8**, anhydrous DMSO, TEA, room temperature (rt), two days (2d).

The synthesis of pullulan conjugates was performed according to multistep protocols, which included the functionalization and activation of the polysaccharide and conjugation of dexamethasone and/or cyanine3 as fluorescent label (Scheme 1).

The NMR, FT-IR and elemental analyses of intermediates showed that the chemical protocols set up for the bioconjugate derivatives yielded the desired products with high yield.

**Figure 1:**
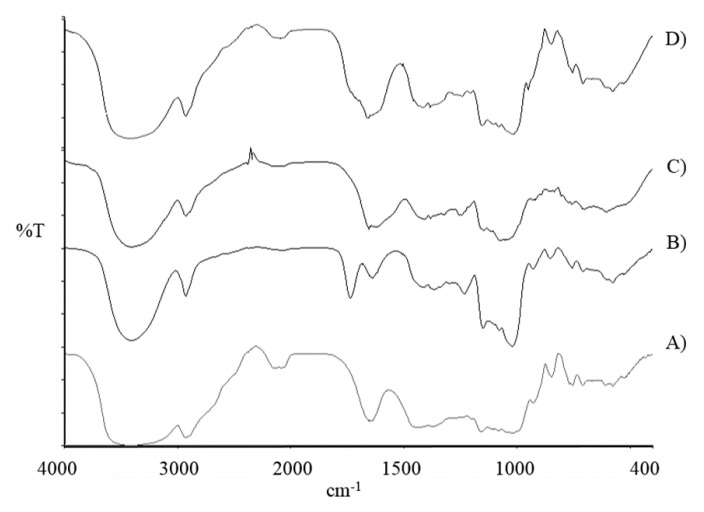
Fourier Transform–Infrared Spectrum of (**A**) pullulan: 3410 (-OH), 2928 (C-H), 1648 (C-O-C) cm^−1^; (**B**) carboxyethyl-pullulan: 3402 (-OH), 2930 (C-H), 1736 (O-C=O), 1638 (C-O-C) cm^−1^; (**C**) carboxyhydrazide-pullulan: 3400 (-OH), 2929 (C-H), 1655 (N-C=O), 1638 (C-O-C) cm^−1^; (**D**) pullulan-dexamethasone: 3408 (-OH), 2926 (C-H), 1654 (-C=O-NH-N=C), 1637 (C-O-C) cm^−1^. %T = Transmittance in percentage.

The FT-IR spectra reported in Figure 1 show in all pullulan derivatives the absorption band around 1648 (C-O-C) cm^−1^ of the polysaccharide. The signal at 1736 cm^−1^ of the carboxyethyl-pullulan in Figure 1B corresponds to the keto group (C=O) of carboxyethyl moiety, confirming the activation and conjugation of carboxyethyl group to the pullulan backbone. After conversion of the carboxyethyl groups of pullulan to hydrazides (-C=O-NHNH_2_), the signal at 1736 cm^−1^ of the FT-IR spectrum attributed to the carboxylic group (-O-C=O-) shifted to 1655 cm^−1^ (Figure 1C). The conjugation with dexamethasone through hydrazone remained in the shifted signal at around 1654 cm^−1^ attributed to the hydrazones (-C=O-NH-N=C) (Figure 1D).

**Figure 2:**
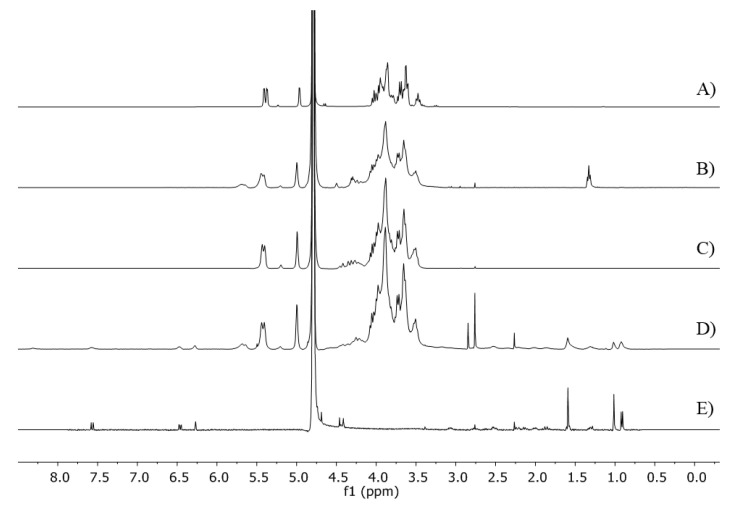
^1^H NMR (400 MHz) spectra of (**A**) pullulan, (**B**) carboxyethyl-pullulan, (**C**) carboxyhydrazide-pullulan, (**D**) pullulan-dexamethasone and (**E**) dexamethasone in D_2_O.

The ^1^H NMR spectra reported in Figure 2A–D show the characteristic proton signals of anomeric carbon of pullulan maltotriose units, consisting of (1→4)-α-glycosidic bond (5.45 ppm) followed by (1→6)-α-glycosidic bond (5 ppm). The broader signals in the pullulan derivatives are in agreement with the modification of the polysaccharide backbone. The spectrum of carboxyethyl-pullulan reported in Figure 2B shows the characteristic signal at 1.33 ppm of terminal methyl groups (-CH_3_) of the newly introduced carboxyethyl moiety (-(C=O)-OCH_2_CH_3_) to the primary hydroxyl groups of the saccharides, which confirms successful carboxyethylation of those groups. The degree of glucopyranose unit (GPU) derivatization of carboxyethyl-pullulan was calculated according to the ratio of signals at 5.5 ppm and 1.33 ppm, integrals 1 and 0.68, (details in SI-2), which showed that about 15% of GPU units were modified by carboxyethyl groups (-(C=O)-OCH_2_CH_3_). The ^13^C NMR analysis confirmed the identity of the pullulan intermediate (SI-6, Figure S3). The signals at 172.32, 80.11, 68.43 and 13.31 ppm are attributed to carbons of the carboxyethyl groups (-O-CH_2_-(C=O)-OCH_2_CH_3_) conjugated to pullulan backbone.

The ^1^H NMR analysis of carboxyhydrazide-pullulan reported in Figure 2C shows that the ethoxy groups (-OCH_2_CH_3_) of the carboxyethyl groups were quantitatively converted in the second step to hydrazide groups (-NHNH_2_), yielding the carboxyhydrazide-pullulan derivative. Indeed, the signal at 1.33 ppm attributed to the terminal methyl group of -(C=O)-OCH_2_CH_3_ completely disappeared. Similarly, the ^13^C NMR spectrum (SI-6, Figure S4) showed signals at 170.84 and 80.24 ppm, which correspond to the carbons of the keto-methoxy unit (-O-CH_2_-(C=O)-NHNH_2_) connecting the hydrazide to the pullulan backbone, while the signals of ethoxy groups 68.43 and 13.31 ppm were not detected.

The experimentally calculated carbon, hydrogen and nitrogen contents obtained by elemental analyses of pullulan, carboxyethyl-pullulan and carboxyhydrazide-pullulan matched with the theoretical data. The GPU modification with hydrazide, based on elemental analysis, resulted in 14.8%, which was in agreement with the calculation derived from the NMR analysis (15% GPU modification). Similarly, the colorimetric TNBS assay used to assess the hydrazide content [25] confirmed the conversion of the ethoxy groups (-OCH_2_CH_3_) into hydrazide groups (-NHNH_2_), yielding 14.8% GPU modification. The RP-HPLC analysis of the pullulan-dexamethasone derivative showed that no free drug was present in the final product (SI-7, Figure S5).

The dexamethasone conjugation to the carboxyhydrazide-pullulan backbone was found to slowly take place over time. The degree of conjugation was assessed by RP-HPLC to evaluate the unconjugated drug, and by TNBS to evaluate the disappearance of hydrazide groups (SI-3, Figure S1). After 48 h, the amount of hydrazide groups derivatized with dexamethasone was about 35%. This result was confirmed by ^1^H NMR analysis. The spectrum reported in Figure 2D shows both the characteristic signals of dexamethasone (7.57, 6.47, 6.27 ppm of aromatic protons and 1.59, 1.02, 0.92 ppm of methyl protons) and pullulan. The conjugation degree was calculated by using an internal standard (IS, 4-chloro-3-nitrobenzoic acid). The ratio between the integral of internal standard signals at 8.47, 8.16 and 7.88 ppm attributed to the aromatic protons (1H*ar* IS) and the integral of signals in the range 1.17–0.74 ppm attributed to six hydrogens (two -CH_3_ groups) of conjugated dexamethasone in pullulan-dexamethasone normalized to the pullulan concentration yielded 5.2% of GPU modification with dexamethasone, which corresponds to 10% *w*/*w* conjugation yield of dexamethasone in the conjugate. After purification, no free dexamethasone was found by RP-HPLC analysis (SI-7, Figure S5). Dexamethasone contains two carbonyl groups that can be potentially conjugated to the polysaccharide through hydrazone bond [21]. However, several studies reported in the literature report that the conjugation reaction preferentially occurs at the non-aromatic carbonyl group [22,23,24,25]. This is mainly due to the steric hindrance and electron distribution of the aromatic moiety, which make the aromatic carbonyl group poorly reactive. Interestingly, in the case of dexamethasone conjugation to pullulan reported here, the surrounding protons on the aromatic ring did not significantly change their shifts in the ^1^H NMR spectra, which indicates that the carbonyl group on the aromatic ring is not directly involved in the conjugation.

### 3.2. Colloidal Characterization

**Figure 3:**
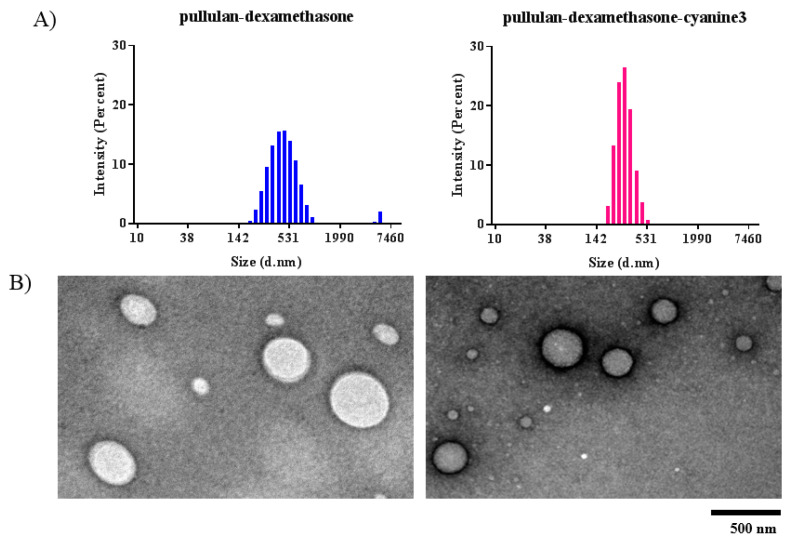
(**A**) Size distribution profile by intensity of pullulan-dexamethasone (■) and pullulan-dexamethasone-cyanine3 (■). (**B**) TEM images of pullulan-dexamethasone (**left**) and pullulan-dexamethasone-cyanine3 (**right**). Bar size: 500 nm.

The dynamic light scattering (DLS) profiles reported in Figure 3A show that in mQ water, pullulan-dexamethasone self-assembles to generate particles with an average size of 461 ± 30 nm (PDI 0.39 ± 0.04), while pullulan-dexamethasone-cyanine3 forms smaller and narrower polydisperse nanoparticles (299 ± 42 nm, PDI 0.22 ± 0.11). The transmission electron microscopy (TEM) images show that the particles of pullulan-dexamethasone have an average size of 402 ± 66 nm and those of fluorescently labeled pullulan-dexamethasone-cyanine3 have an average size of 242 ± 61 nm. The slightly smaller size values obtained by TEM analysis with respect to those obtained by DLS may be ascribable to the dry conditions of samples undergoing TEM analysis. The different size of nanoparticles is ascribable to the different composition of the bioconjugates. Indeed, pullulan-dexamethasone-cyanine3 contains 1.1% of cyanine3 in addition of 5.2% GPU derivatized with dexamethasone. Cyanine3 is a hydrophobic molecule that contributes to the association process and intraparticle interactions, which results in smaller size and lower PDI compared to the nanoparticles obtained with pullulan-dexamethasone.

The pullulan-dexamethasone and fluorescently labeled pullulan-dexamethasone-cyanine3 possess negative zeta potentials of −38 and −20 mV, respectively, which is in agreement with results reported in the literature on polysaccharide-based nanoparticles [28,29].

**Figure 4:**
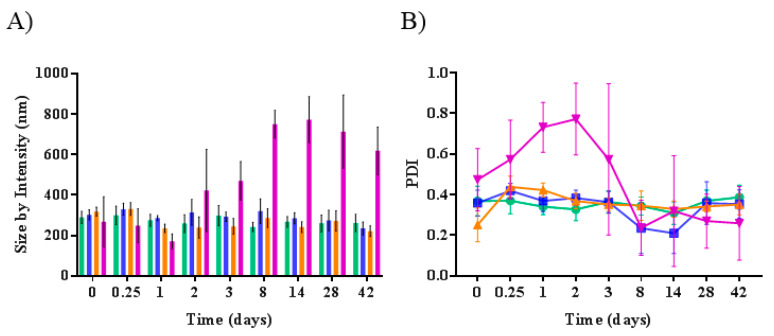
Size (**A**) and poly dispersity index (PDI) (**B**) of pullulan-dexamethasone particles after different exposure times in buffer solution at 4 °C (■), 25 °C (■), 37 °C (■) and in homogenized vitreous at 37 °C (■).

The colloidal stability profiles reported in Figure 4 show that in buffer solution (10 mM phosphate buffer, 150 mM NaCl, pH 7.4), pullulan-dexamethasone nanoparticles were fairly stable over 6 weeks in physiological buffer at storage temperature (4 and 25 °C) and body temperature (37 °C). No significant change was observed either in mean size or PDI. It should be noted that in buffer, the nanoparticles exhibited smaller size and lower PDI as compared to the values obtained in water, suggesting that ionic force and pH may affect the intraparticle interactions. Contrary to buffer, in homogenized vitreous at 37 °C, the colloidal assemblies underwent structural reorganization and physical change over time (at day 0/37 °C and at day 42/37 °C). Throughout the first day of incubation, the mean size of the particles was stable, but from the second day of incubation, both the size and PDI increased, indicating that a rearrangement of the assemblies was taking place, suggesting that the vitreous components, hyaluronic acid, collagen and enzymes may induce colloidal instability with formation of homogeneous large aggregates.

### 3.3. Dexamethasone Release

**Figure 5:**
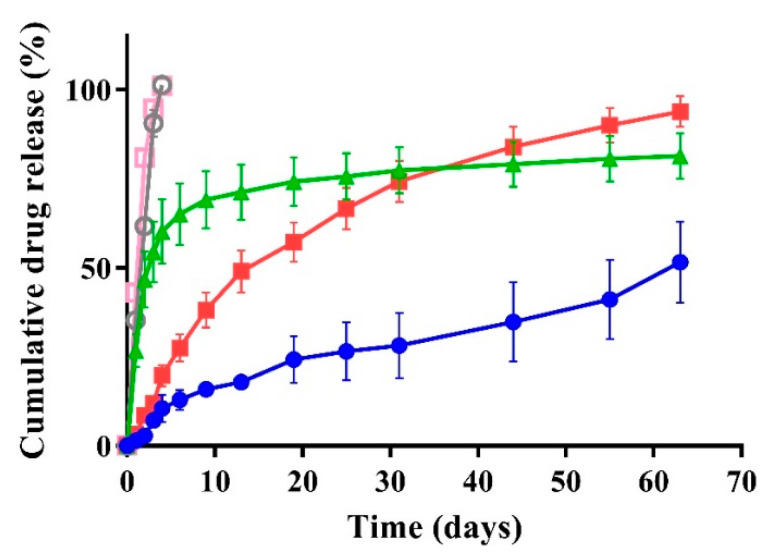
Release profile of dexamethasone from pullulan-dexamethasone under three different conditions: PBS pH 7.4 (●); 1:1 *v*/*v* homogenized vitreous: PBS, pH 7.4 (■); phosphate-citric buffer, pH 5.0 (▲). Free dexamethasone in PBS, pH 7.4 (○) and in 1:1 *v*/*v* homogenized vitreous:PBS, pH 7.4 (**□**).

Figure 5 reports the dexamethasone release profiles under conditions mimicking vitreous and lysosomal compartment. The chromatographic and ESI-TOF mass spectrometry analyses confirmed that the drug was released in its native form (SI-7, Figures S6 and S7). This result is in agreement with previously published data showing that in the case of hydrazone-linked bioconjugates, the cleavage of the hydrazone bond is the main degradation process [18]. However, the previous study showed also that additional chemical cleavages can contribute to the backbone degradation, while in vivo enzymes could participate in the overall polymer degradation.

In physiological buffer at pH 5.0, a burst release of about 70% was seen over one week, and 50% of the drug was released in about 2 days (Figure 5). This is in line with the low stability of the hydrazone bond under acidic conditions. Therefore, in the case of the bioconjugate localization in the lysosomes the drug would be easily released in this compartment. After one week, a slow increase of free dexamethasone concentration was observed over the following 8 weeks. During this time the drug could be partially degraded because of its limited stability under acidic conditions [31].

In physiological buffer at pH 7.4, 50% of the drug was released following a nearly linear profile over 9 weeks, which is in agreement with the higher stability of the hydrazone bond at nearly neutral pH compared to acidic conditions. In homogenized vitreous, the release of dexamethasone was faster compared to the one in PBS at pH 7.4; 50% of the drug was released in about 2 weeks. We may speculate that the enzymatic activity in the vitreous [32,33,34] increases the cleavage of dexamethasone from the pullulan backbone. Anyway, dexamethasone release took place in about 2 months in the presence of vitreous.

### 3.4. In Vitro Biocompatibility and Cell Association Studies

**Figure 6:**
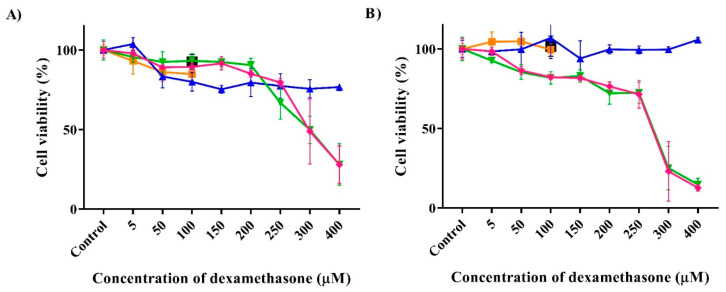
ARPE-19 cell viability profiles by MTT assays (**A**) after 24 h incubation with samples, and (**B**) after 24 h incubation followed by removal of samples and 48 h cell proliferation. Control are untreated cells (negative control, no polymeric conjugate or drug added), DMSO in the medium (0.8% *v*/*v*, one black squared symbol, ■), dexamethasone (positive control, ■) and carboxyhydrazide-pullulan as drug-free polymer (▲), pullulan-dexamethasone (▼) and fluorescently labeled pullulan-dexamethasone-cyanine3 (◆). The concentration of free dexamethasone or equivalent conjugated dexamethasone in pullulan-dexamethasone is plotted. The concentration of the carboxyhydrazide-pullulan was equivalent to that of pullulan in the cell samples treated with pullulan-dexamethasone.

Cell toxicity studies were performed with increasing concentrations of dexamethasone-free carboxyhydrazide-pullulan, pullulan-dexamethasone, fluorescently labeled pullulan-dexamethasone-cyanine3 and dexamethasone. Due to its poor water solubility (40 µg/mL), dexamethasone was used in this study in a concentration range of 0–100 μM in DMEM/F12 medium. The cell viability profile at increasing equivalent dexamethasone concentration is shown in Figure 6.

When cell studies were performed for 24 h, the pullulan-dexamethasone and pullulan-dexamethasone-cyanine3 nanoparticles showed a significant cytotoxicity above 200 μM equivalent dexamethasone concentrations (Figure 6A), while below 200 μM the slight cytotoxicity was similar to that observed with the free drug or the polymeric scaffold. The well-tolerated carboxyhydrazide-pullulan shows that the presence of hydrazide groups in pullulan does not result in a toxic polymer and indicates that hydrazide moieties re-established after dexamethasone release do not induce cytotoxicity. Similar behavior was observed when the cell viability was examined after 48 h post-incubation with DMEM/F12 medium (Figure 6B). However, in this case the dexamethasone conjugates displayed slightly higher toxicity with respect to the previous experiment, which may be due to higher cellular uptake of the polymer conjugates and slow dexamethasone release in the cells. To investigate the cell uptake of the polymeric derivatives, flow cytometric analyses (FACS) were performed with ARPE-19 cell line and pullulan-dexamethasone-cyanine3.

**Figure 7:**
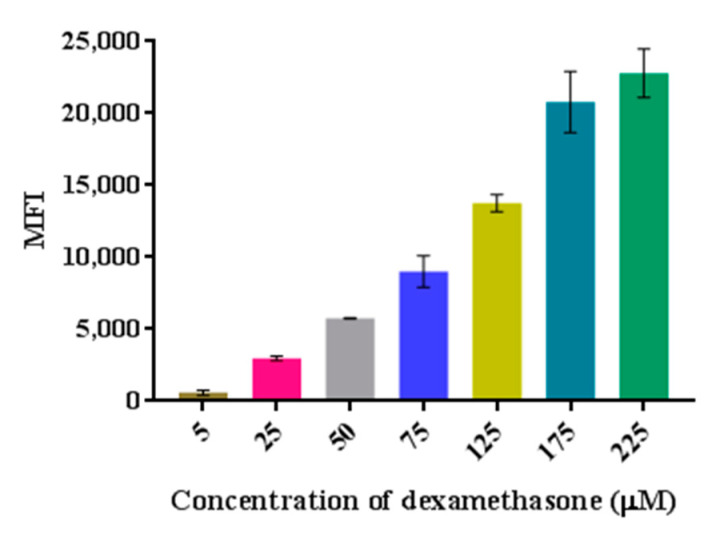
Flow cytometric profile of ARPE-19 cells incubated with pullulan-dexamethasone-cyanine3 at increasing concentration. Concentration is referred to conjugated dexamethasone. MFI: Mean Fluorescence Intensity.

The results in Figure 7 show that, as expected, the cellular association of the particles increased with increasing conjugate concentration.

These results show that, even though the pullulan-based nanoparticles have negative zeta potential, which would set up an electrostatic repulsion with the cell membrane, they interact very efficiently with the cells.

**Figure 8:**
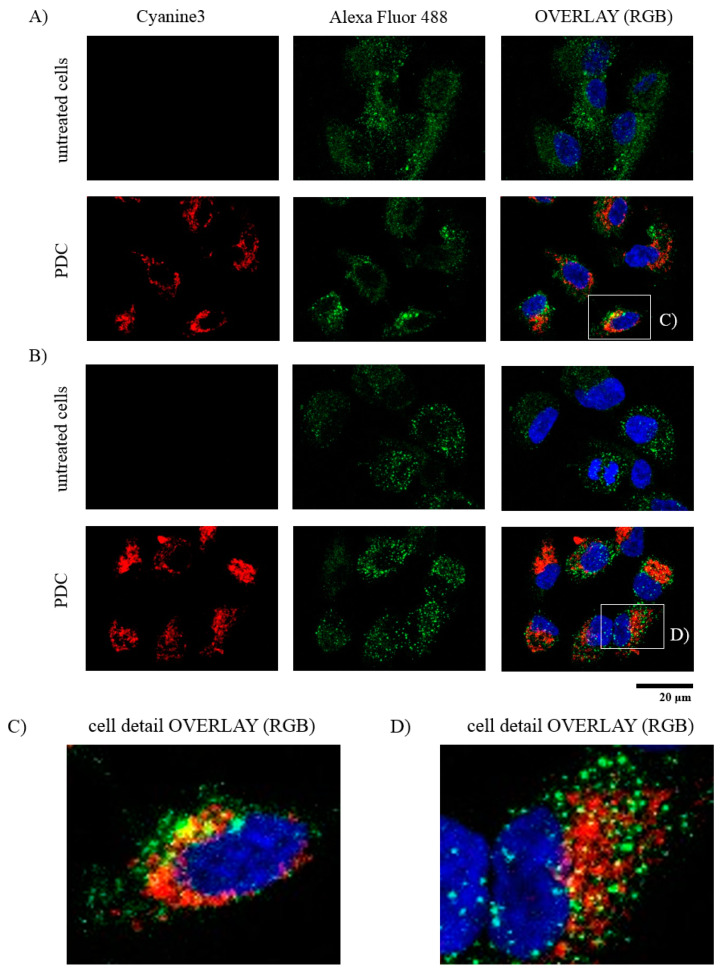
Confocal microscopic images of ARPE-19 cells incubated with 0.2 mg/mL of pullulan-dexamethasone-cyanine3 (PDC) nanoparticles in red. (**A**) Endosomes were stained in green with rabbit anti-EEA1 and Alexa Fluor 488 labeled goat anti-rabbit IgG H&L secondary antibody. (**B**) Lysosomes were stained in green with rat anti-mouse anti-LAMP1 and Alexa Fluor 488 labeled goat anti-rat IgG H&L secondary antibody. Nuclei were stained with DAPI in blue. RGB: overlay of red, green and blue channels for organelles/nanoparticles co-localization. (**C**) Magnification of the cell in the white square from (**A**) endosomes/nanoparticles from overlay (RGB). (**D**) Magnification of the cell in the white square from (**B**) lysosomes/nanoparticles from overlay (RGB). Bar size: 20 μm. (Note: bright field; RG: overlay of red and green channels are in SI-8, Figure S8).

To assess if the pullulan-dexamethasone nanoparticles are simply adsorbed on the cell membrane or are endocytosed by the cells, confocal laser scanning microscopic imaging was performed. Based on the fluorescence of the samples detected by the cytofluorimetric analysis, the concentration 0.2 mg/mL of pullulan-dexamethasone-cyanine3 nanoparticles, corresponding to 25 μM equivalent dexamethasone concentration, was selected for the confocal imaging. Nanoparticle localization in ARPE-19 cell endosomes and lysosomes was investigated to obtain information about the cell trafficking by labeling endosomes with rabbit anti-EEA1 and Alexa Fluor 488 labeled goat anti-rabbit IgG and lysosomes with rat anti-mouse anti-LAMP1 and Alexa Fluor 488 labeled goat anti-rat IgG. Figure 8A,C show that pullulan-dexamethasone-cyanine3 nanoparticles strongly associate with the cells and localize within endosomes as detected by the fluorescence overlay of the cyanine3 labeled conjugate and the Alexa Fluor 488 labeled antibody used for endosome staining. The images in Figure 8B,D show that no pullulan-dexamethasone-cyanine3 localization was observed in lysosomes. Indeed, fluorescence of the conjugate (red) and Alexa Fluor 488 labeled antibodies (green) did not overlap. These data indicate that the conjugates are confined within endosomes where, according to the release profiles obtained at different pH, the drug could be released over a longer time than in lysosomes. However, it should be noted that images were collected by using cells incubated for 1, 3 and 5 h with the conjugate, which may not be a sufficient time for full lysosomal localization.

As a small lipophilic molecule, the released dexamethasone is expected to escape from endosomes and lysosomes and reach cytosol and nucleus. Enzymatic activity in the cells and acidic pH of the endosomal and lysosomal compartments may enhance localized dexamethasone release from pullulan conjugates in the cells, in analogy with the previously published peptidic delivery system [35]. Thus, intravitreally injected pullulan conjugates may release dexamethasone both in the vitreous and within the ocular cells after internalization of the polymer conjugate. Thus, polymeric conjugates may enable synergistically prolonged retention, controlled release, and localized intracellular drug release.

## 4. Conclusions

Pullulan is a promising carrier for drug conjugation that has not been tested for ocular drug delivery. We developed a robust synthetic procedure for conjugation of drugs possessing either ketone or aldehyde functional groups to pullulan for controlled release. Dexamethasone was selected since it possesses a ketone group and, in virtue of its hydrophobic character, can participate to yield an amphiphilic conjugate with self-assembling features. Synthetic procedures involved derivatization of pullulan with hydrazide and further conjugation of dexamethasone. The bioconjugates formed self-assembled nanoparticles that released dexamethasone at a slow rate in the vitreous, while the drug is released at a significantly faster rate at endosomal and lysosomal pH 5. The pullulan-based nanoparticles undergo retinal ARPE-19 cell association and internalization. Thus, the drug conjugation to pullulan may result in prolonged drug delivery to the target tissue based on the obtained data showing slow drug release, negative charge, small size, cellular uptake and safety. These results are encouraging for further retinal ex vivo and in vivo studies.

## Data Availability

The data presented in this study are available on request from the corresponding author.

## Supplementary Materials

The following are available online at https://www.mdpi.com/article/10.3390/pharmaceutics13060791/s1, SI-1: Characterization of pullulan; SI-2: ^1^H NMR analysis of pullulan conversion to carboxyethyl-pullulan; SI-3 and Figure S1: Dexamethasone conjugation rate., SI-4 ^1^H NMR analysis of dexamethasone conjugation to carboxyethyl-pullulan; SI-5 and Figure S2: Dexamethasone release; SI-6, Figures S3–S4: ^13^C NMR spectra or carboxyethyl-pullulan and carboxyhydrazide-pullulan; SI-7 and Figures S5–S7: Dexamethasone conjugation; SI-8 and Figure S8: Confocal microscopy.
