# Supplementary material for: Pullulan Based Bioconjugates for Ocular Dexamethasone Delivery
Source: Pharmaceutics. 2021 May 26;13(6):791. doi: 10.3390/pharmaceutics13060791 (PMC8227697; doi:10.3390/pharmaceutics13060791)
Supplement: Supplementary file 1 [file pharmaceutics-13-00791-s001.zip › pharmaceutics-1215725-supplementary.pdf]

pharmaceutics

Supporting information: Pullulan Based Bioconjugates for Ocular Dexamethasone Delivery

Eva Kicková, Stefano Salmaso, Francesca Mastrotto, Paolo Caliceti and Arto Urtti

SI-1 Characterization of pullulan

FT-IR (KBr). 3410 (-OH), 2928 (C-H), 1648 (C-O-C) cm^{-1}.

Elemental analysis. Calcd: C, 43.90%; H, 6.28%. Found: C, 40.32%; H, 6.38%.^1H NMR (600 MHz, D_2O). δ 5.40 (dd, 1H, (1→4)-α-glycosidic bond), 4.98 (d, 1H, (1→6)-α-glycosidic bond), 4.09-3.37 (5H, remaining Hs of glucopyranose).

^{13}C NMR (151 MHz, D_2O). δ 100.19, 99.74, 97.89, 95.86, 92.05, 77.74, 77.36, 75.90, 75.65, 74.09, 73.41, 73.31, 73.02, 72.71, 71.68, 71.59, 71.48, 71.39, 71.31, 71.13, 70.78, 70.30, 69.60, 69.55, 69.45, 69.22, 66.46, 63.17, 60.68, 60.54, 60.37.

Data are in accordance with the literature [1–3].

SI-2 ^1H NMR analysis of pullulan conversion to carboxyethyl-pullulan

Conversion yield of pullulan carboxyethyl-pullulan was measured by ^1H NMR spectroscopy in deuterated water (D_2O) according to the general equation (1) [1]:

(integral value of selected protons in conjugated group/number of protons of that conjugated group) *66 (1)

Integration of signal area at 5.45 ppm was set as 1 (1H, (1→4)-α-glycosidic bond). The integral of signal at 1.33 ppm corresponding to carboxyethyl group (-CH_3) was 0.68 which was divided by 3 (3 protons from -CH_3 of carboxyethyl group), multiply by 66 [66 is the number of protons (1H) associated to the (1→4)-α-glycosidic bond refered to 100 glucose monomers].

In order to calculate the GPU percentage modification with carboxyethyl groups: (0.68/3) *66=15% of the GPU modification by carboxyethyl groups in pullulan in the carboxyethyl-pullulan.

SI-3 Dexamethasone conjugation rate

The dexamethasone conjugation to carboxyhydrazide-pullulan over reaction time was assessed by detecting the disappearance of free dexamethasone by RP-HPLC and hydrazyde groups by TNBS. Figure S1 shows the dexamethasone conjugation profile to the polysaccharide. The reaction was terminated when the conjugation yield of dexamethasone was in the 5.0-5.5% GPU range.

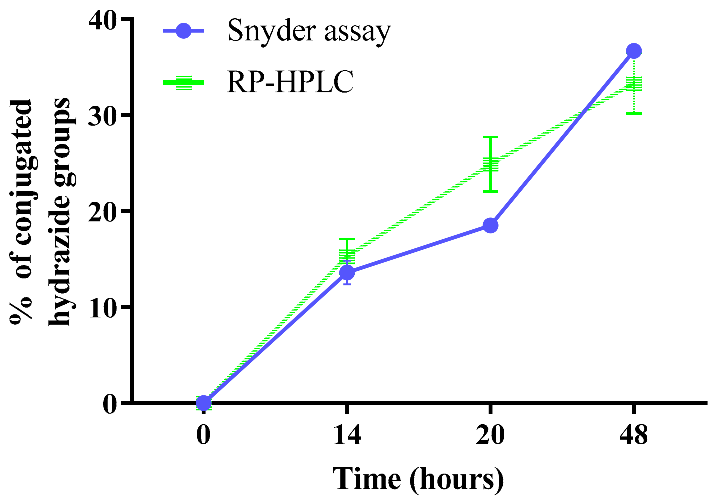

Figure S1. Kinetic progress of the conjugation between dexamethasone and carboxyhydrazide-pullulan by RP-HPLC analysis (■) and by TNBS assay (●).

SI-4 ^{1}H NMR analysis of dexamethasone conjugation to carboxyethyl-pullulan

^{1}H NMR spectroscopic method was used to derive the pullulan conversion yield in pullulan-dexamethasone, pullulan-dexamethasone-cyanine3 and pullulan-cyanine3. The analysis was performed in DMSO-d_6 with an internal standard (4-chloro-3-nitrobenzoic acid) and the following equation was then applied:

[Integral of signal of internal standard: Integral of signal of selected protons of sample] = [number of mmol IS: number of mmol in selected sample] (2)

The internal standard integrals of signal at known molarity was used to calculated the molarity of dexamethasone in the conjugate pullulan-dexamethasone. Based on the weighed amount of conjugate for the analysis, it was possible to then derive the dexamethasone % w/w in the conjugate and then the GPU units modified with dexamethasone with respect to total GPU.

SI-5 Dexamethasone release

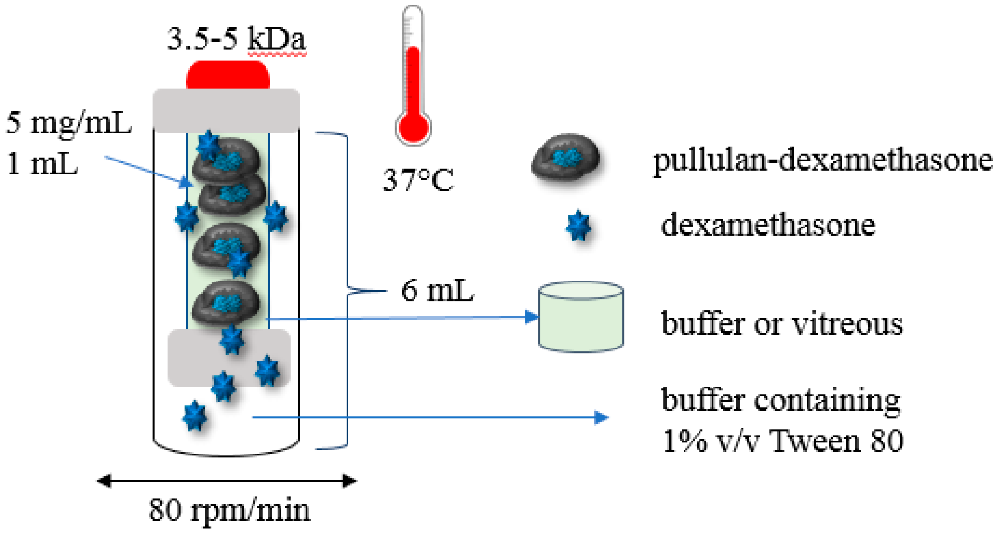

Figure S2. Schematic representation of the set up for the dexamethasone release by pullulan-dexamethasone under physiologically simulating conditions.

Dexamethasone solubility was assessed in 1% v/v Tween 80 aqueous solution.

A dexamethasone suspension (1.5 mg/mL) was prepared in PBS, pH 7.4, containing 1% v/v Tween 80. The sample was incubated at room temperature with orbital shaking at 100 rpm/min for 20 h in the dark. The suspension was centrifuged at 4000 rpm/min for 20 min and then 1 mL of the surnatant was transferred in a microtube and centrifuged again three times at 14,000 rpm/min for 10 min. A volume of 10 μL of surnatant was diluted with 1.49 mL of 0.1 M HCl containing 1% v/v Tween 80. The diluted samples were analysed by RP-HPLC. The solubility of dexamethasone in PBS, pH 7.4, containing 1% v/v Tween 80 was found to be 0.2 mg/mL.

SI-6 ^{13}C NMR spectra of carboxyethyl-pullulan and carboxyhydrazide-pullulan

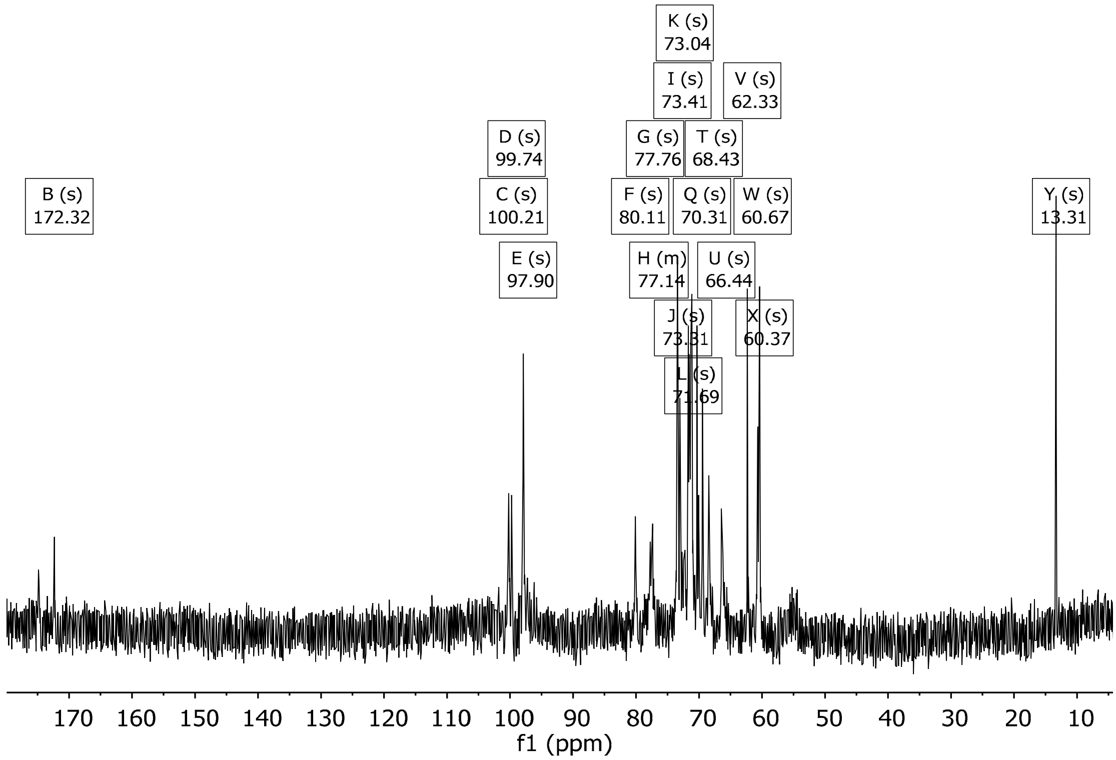

Figure S3. The ^{13}C NMR spectrum of carboxyethyl-pullulan in D_2O.

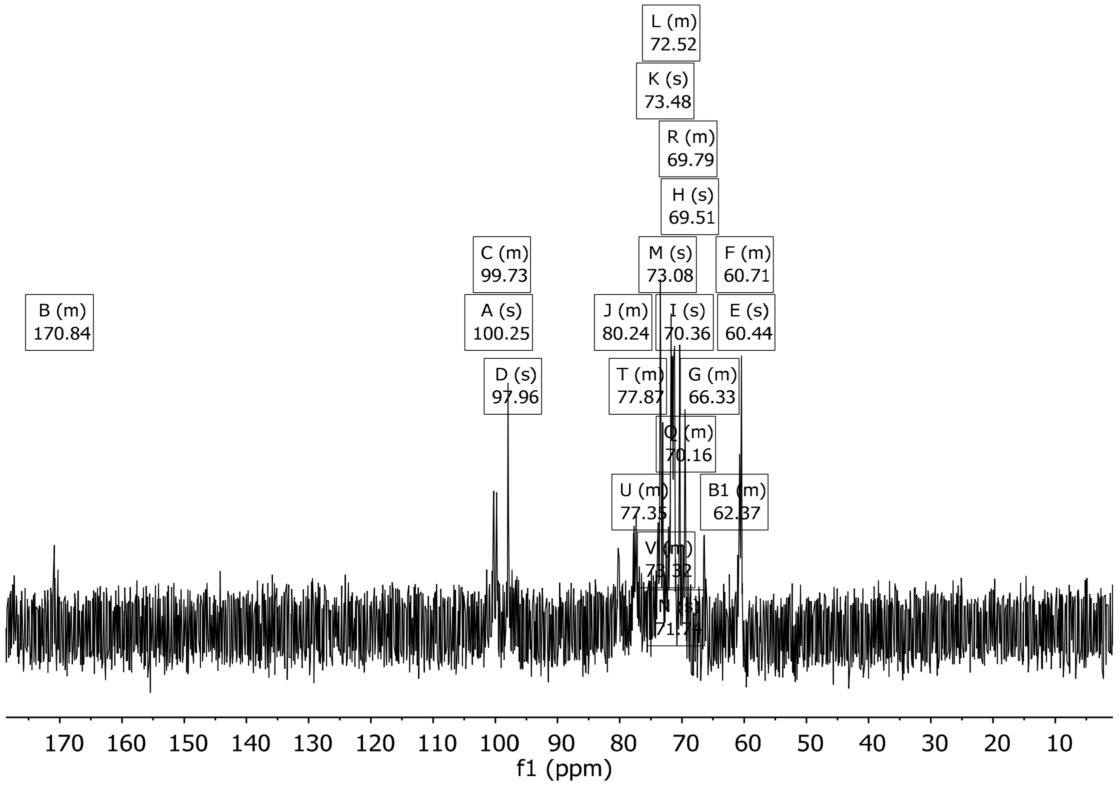

Figure S4. The ^{13}C NMR spectrum of carboxyhydrazide-pullulan in D_2O.

SI-7 Dexamethasone conjugation

To confirm the efficiency of purification after conjugation of dexamethasone to carboxyhydrazide-pullulan, the presence of free unconjugated dexamethasone in the pullulan-dexamethasone conjugate was assessed with RP-HPLC analysis (Figure S5). The sample was dissolved in PBS, pH 7.4 with 1% v/v Tween 80 (5 mg/mL) and immediately analysed to prevent release of conjugated dexamethasone. No peak of eluted free dexamethasone **(**retention time = 10.61 min.) was detected on the chromatographic profile. This analysis confirmed the efficiency of the purification method and the purity of the pullulan-dexamethasone.

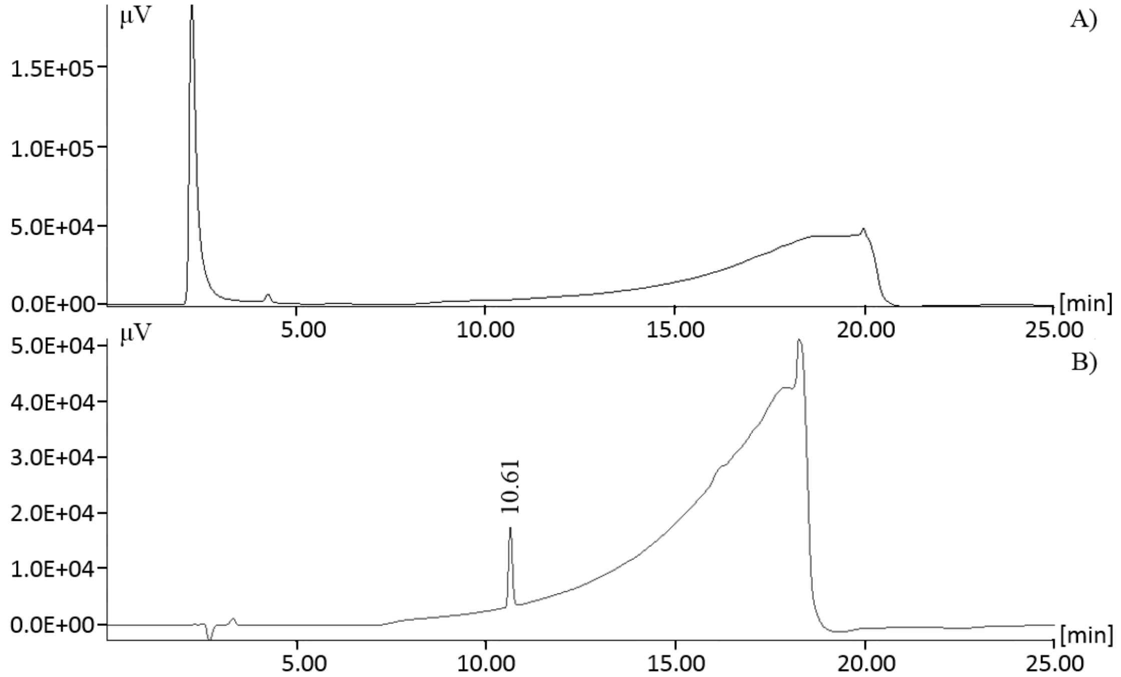

Figure S5. RP-HPLC profiles of A) pullulan-dexamethasone after synthesis and purification, and B) free dexamethasone (retention time: 10.61 min).

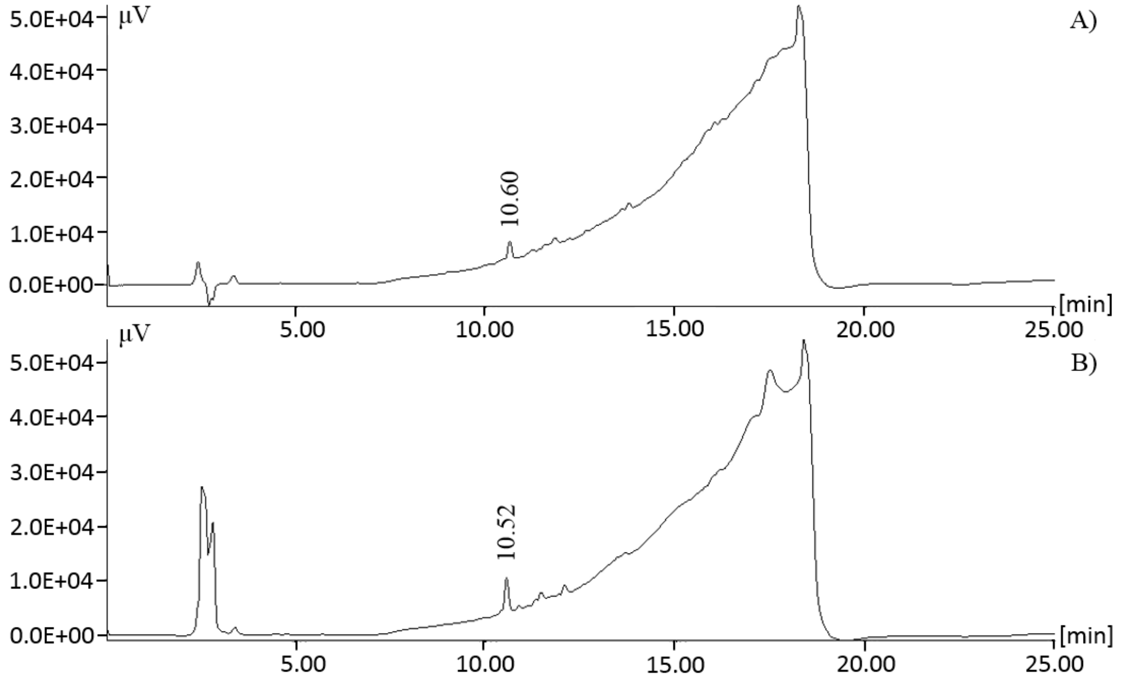

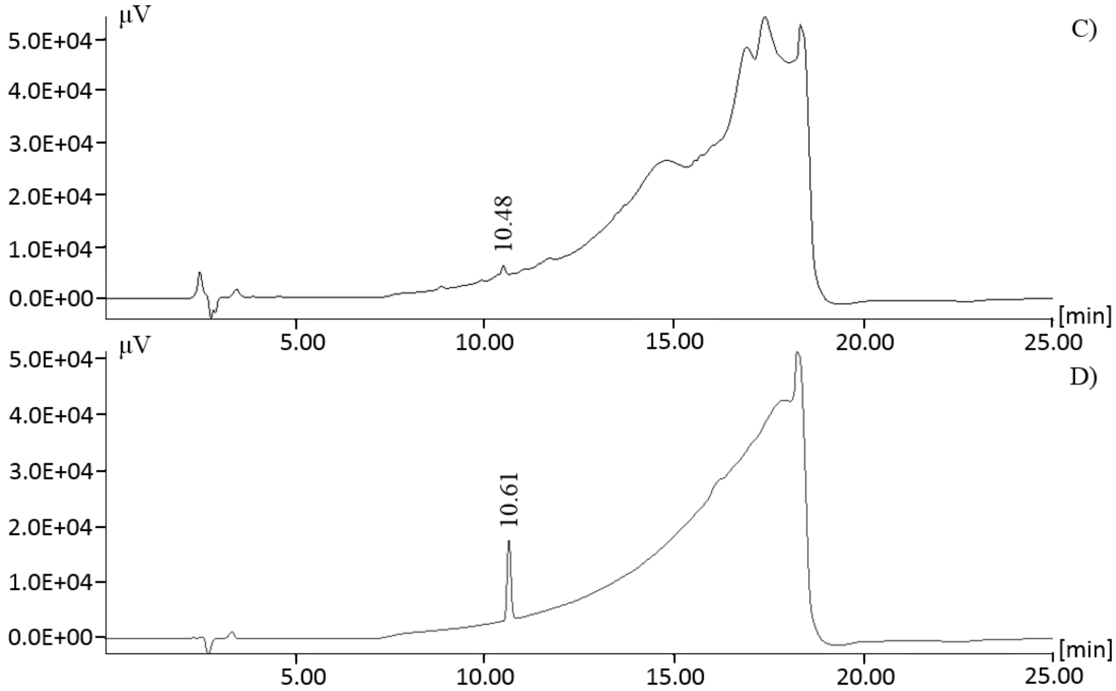

Figure S6. RP-HPLC profiles of released dexamethasone from pullulan-dexamethasone in different pH conditions: A) PBS, pH 7.4; B) 10 mM phosphate, 24 mM citric acid, 150 mM NaCl, pH 5; C) 1:1 v/v homogenized vitreous:PBS, pH 7.4; D) free dexamethasone in PBS, pH 7.4. The chromatographic profiles correspond to samples analyzed after 4 days of release.

The experimental spectrum of the released dexamethasone (Figure S7A) in positive ionization shows a m/z of 393.2075 ([M+H]+) which is in agreement with the expected mass spectrum in positive ionization (m/z calculated 393.2077) of dexamethasone (Figure S7B).

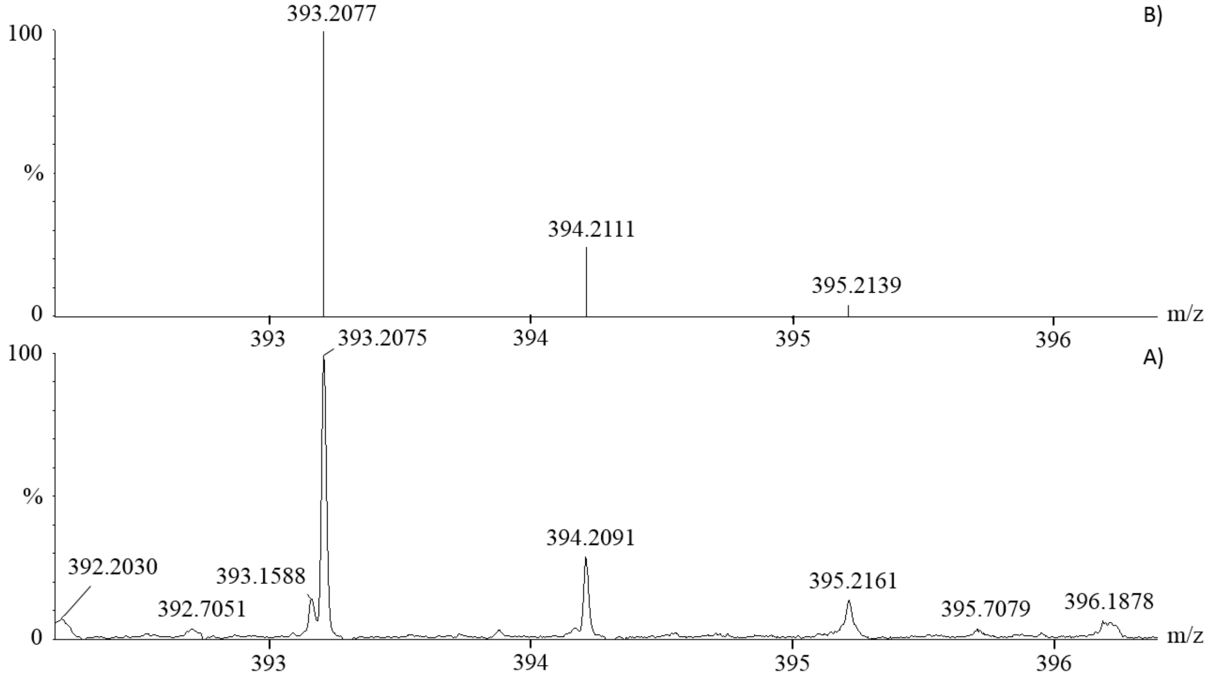

Figure S7. ESI-Tof mass spectrum profiles of dexamethasone in positive ionization: A) experimental mass spectrum; B) expected mass spectrum. The experimental mass spectrum was performed with drug released by pullulan-dexamethasone after 19 days.

SI-8 Confocal microscopy

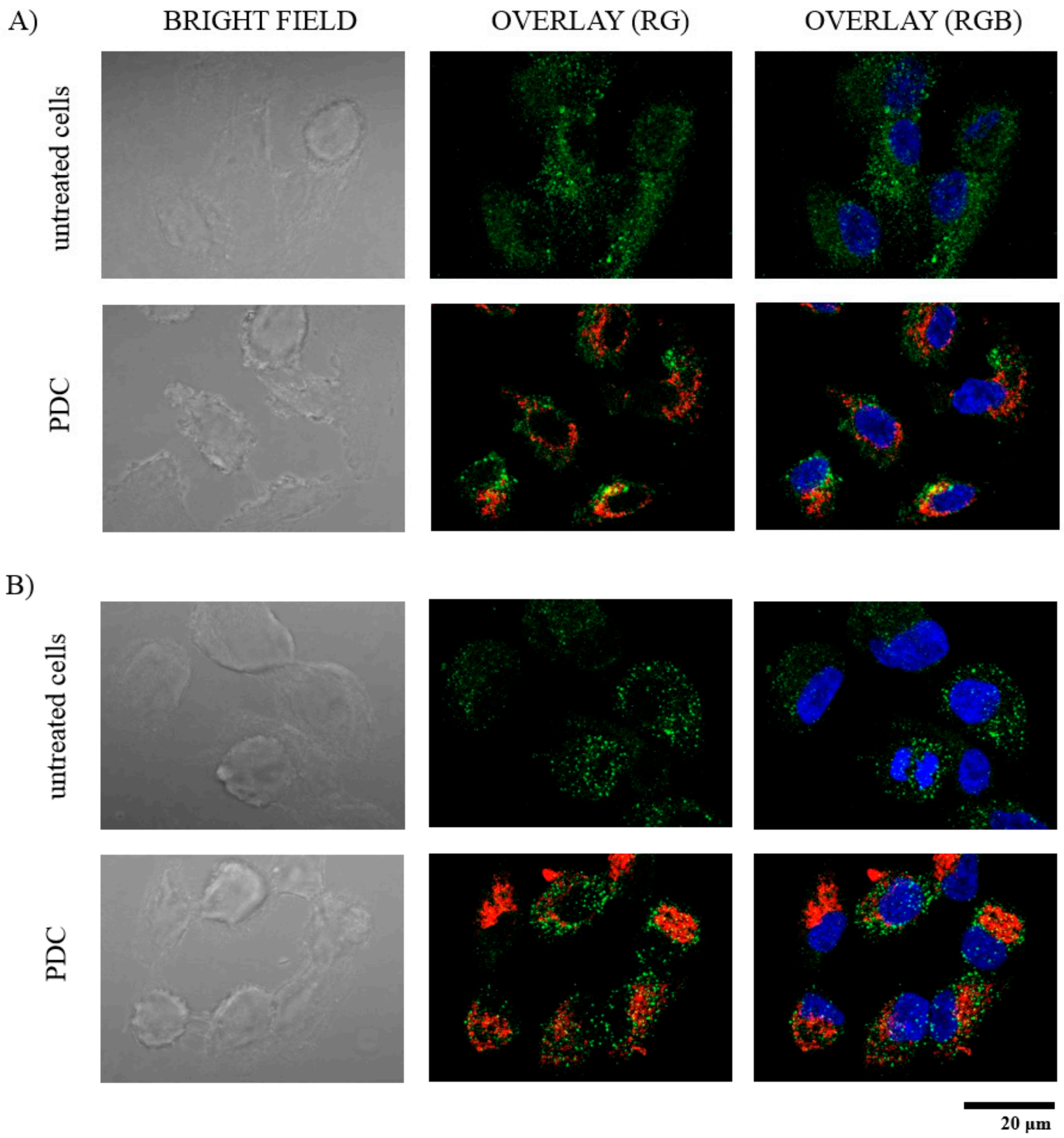

Figure S8. Confocal microscopic images of ARPE-19 cells incubated with 0.2 mg/mL of pullulan-dexamethasone-cyanine3 (PDC) nanoparticles in red with bright fields. (A) Endosomes were stained in green with rabbit anti-EEA1 and Alexa Fluor 488 labeled goat anti-rabbit IgG H&L secondary antibody. (B) Lysosomes were stained in green with rat anti-mouse anti-LAMP1 and Alexa Fluor 488 labeled goat anti-rat IgG H&L secondary antibody. Nuclei were stained with DAPI in blue. RG: overlay of red and green channels and RGB: overlay of red, green and blue channels for organelles/nanoparticles co-localization. Bar size: 20 µm.

References

[1] F. Hassanzadeh, J. Varshosaz, G.A. Khodarahmi, M. Rostami, F. Hassanzadeh, Biotin-encoded pullulan-retinoic acid engineered nanomicelles: preparation, optimization and in vitro cytotoxicity assessment in MCF-7 cells, Indian J. Pharm. Sci. 78 (2016) 557–565.

[2] T.D. Leathers, Biotechnological production and applications of pullulan, Appl. Microbiol. Biotechnol. 62 (2003) 468–473.

[3] N. Tamura, M. Hirota, T. Saito, A. Isogai, Oxidation of curdlan and other polysaccharides by 4-acetamide-TEMPO/NaClO/NaClO2 under acid conditions, Carbohydr. Polym. 81 (2010) 592–598.

[4] S.L. Snyder, P.Z. Sobocinski, An improved 2, 4, 6-trinitrobenzenesulfonic acid method for the determination of amines, Anal. Biochem. 64 (1975) 284–288.
